# Efficacy and resilience are associated with environmental action: an exploratory study of psychological capital components

**DOI:** 10.3389/fpsyg.2026.1784390

**Published:** 2026-03-27

**Authors:** Jarle Eid, Jessica Tengvall, Katja Enberg

**Affiliations:** 1 Department of Psychosocial Science, University of Bergen, Bergen, Norway; 2 Department of Biological Sciences, University of Bergen, Bergen, Norway

**Keywords:** efficacy, environmental action, focus group, hope, optimism, positive psychology, resilience, sustainability

## Abstract

**Introduction:**

This two-part exploratory study examines whether Psychological Capital (PsyCap) can contribute to understanding individual differences in environmental actions.

**Methods:**

In the first phase, 55 students (ages 21–33) completed the 12-item Psychological Capital Questionnaire (PCQ) and the 18-item Environmental Activities and Action Scale (EAA). One week later, the same participants joined focus groups to discuss how the four PsyCap components—Hope, Efficacy, Resilience, and Optimism—might shape motivations for environmental action.

**Results:**

Quantitative analyses suggested that PsyCap factors explained a modest portion (approximately 26.5%) of the variance in environmental action, with Efficacy and Resilience showing the strongest unique contributions (8.6 and 8.0% of the variance, respectively). These relationships should be interpreted cautiously given the limited sample and the mixed pattern of effects across PsyCap components. The focus group discussions added nuance, indicating that Hope, Efficacy, and Resilience may function as potential motivational catalysts, whereas Optimism could in some cases reduce perceived urgency for environmental action.

**Discussion:**

Although preliminary, this pilot-study highlights possible pathways through which specific psychological resources may relate to environmental action. The findings underscore the need for larger, more rigorous studies to clarify the conditions under which PsyCap supports—or fails to support—environmentally responsible behavior.

## Introduction

The growing number and severity of environmental challenges demand large-scale collective action to effectively address their impact (Dietz et al., 2009; Intergovernmental Panel on Climate Change, 2018; Stern, 2000). However, several barriers hinder the adoption of pro-environmental behaviors, as these actions are often perceived as more costly, time-consuming, and less enjoyable than their environmentally harmful alternatives (Steg et al., 2014; United Nations Economic and Social Commission for Asia and the Pacific, 2024). To overcome these barriers, effective collective action is essential—not only to engage different segments of society but also to influence media narratives and prompt decision-makers to act. Importantly, the relationship between environmental awareness and environmental behavior is neither straightforward nor guaranteed (van Valkengoed et al., 2022). A range of factors has been shown to influence environmentally responsible behavior, including social capital, particularly in the form of community participation and trust. For instance, Atshan et al. (2020) found that dimensions of social capital influence environmentally responsible behavior through environmental concern, with social trust emerging as a significant predictor. Similarly, evidence from Macias and Williams (2016) suggests that strong community ties, as an expression of social capital, increase the likelihood of individuals engaging in private environmental actions. In this pilot study, we examine whether recent developments in work-and-organizational psychology can contribute to explaining environmental action. Specifically, we explore how research on positive organizational behavior (Luthans et al., 2007a,b) and the concept of Psychological Capital (Luthans et al., 2006) can inform our understanding of how individual predispositions and preferences may support environmental activities and actions (Alisat and Riemer, 2015).

A growing body of research has turned its attention to social identity, policy acceptance, and social influence as mechanisms for encouraging public support for environmental protection (Barth et al., 2021). A central aim of social identity research is to understand individuals’ willingness to engage in collective action that promotes sustainable lifestyles and environmental protection (Fielding and Hornsey, 2016). Collective action can take many forms, ranging from low-level civic participation, such as staying informed about environmental issues and attending community events, to high-level political engagement, including organizing protests or leading advocacy efforts. According to social identity theory, group norms and identification are key predictors of pro-environmental behavior (Fritsche et al., 2018; Fritsche and Masson, 2021). These social dynamics shape individuals’ perceptions of environmental responsibility and influence their motivation to act.

An important yet often overlooked dimension of social identity theory in the context of climate action is the role of intrapersonal skills and competencies, commonly referred to as *action competence*, which are essential for effective sustainability change agents (Ayers et al., 2023). Among these, emotions and emotion regulation strategies have emerged as critical pathways for motivating pro-environmental behavior. They highlight the significant role that individual consumers’ behavioral preferences and choices play in climate change mitigation (Kovacs et al., 2024). Beyond intrapersonal capacities, there is growing recognition that successful climate action also depends on interpersonal skills—specifically, the ability to engage, collaborate with, and motivate diverse stakeholders (Brundiers et al., 2021). These intra- and interpersonal competencies align closely with over two decades of research in positive organizational behavior, which has systematically mapped and measured state-like psychological resource capacities relevant to work life and performance (see Luthans and Youssef, 2017 for an overview).

The complexity and potentially devastating consequences of global warming are often perceived as overwhelming and insurmountable. Fear-based messaging, while intended to raise awareness, may inadvertently lead to avoidance, denial, or feelings of helplessness, thereby reducing motivation for action. In contrast, hope-based and optimistic messaging has been shown to be more effective in encouraging action (Brosch, 2021). In the present study, we examine the role of positive messaging by exploring how the intrapersonal resource capacity of Psychological Capital (PsyCap) may foster trust and facilitate environmental action. By focusing on the positive psychological traits of *Hope*, *Efficacy*, *Resilience*, and *Optimism*, we aim to understand how individuals can be empowered to contribute meaningfully to climate change mitigation.

Recent meta-analytic evidence underscores the psychological benefits of pro-environmental behavior. Zawadzki et al. (2020) found that engaging in Pro-Environmental Behavior (PEB) is consistently associated with greater subjective well-being (d + ≈ 0.17), suggesting that sustainable actions can enhance life satisfaction. PsyCap may amplify this effect by fostering hope and optimism, which are known to support well-being and persistence in goal pursuit. At the same time, research on behavioral spillovers highlights the challenge of sustaining multiple pro-environmental behaviors. Maki et al. (2019) reported that initial PEB interventions yield only small positive spillovers in intentions and negligible effects on secondary behaviors. PsyCap components such as resilience and efficacy may counteract this tendency by sustaining motivation and perceived capability across different behaviors. Furthermore, Van Valkengoed et al. (2022) propose a framework linking interventions to behavioral determinants, emphasizing the need for targeted strategies. PsyCap could serve as a cross-cutting determinant within this framework, enabling interventions that build hope and efficacy alongside normative and informational approaches. Finally, identity remains a robust predictor of PEB. Udall et al. (2021) show that identity strongly correlates with PEB, particularly when behavior and identity match. PsyCap may interact with identity processes by providing intrapersonal resources—such as hope and resilience—that enable individuals to enact group norms and sustain collective action. These insights position PsyCap as a potentially relevant construct within multi-level models of environmental action.

Positive organizational behavior (Luthans, 2002; Luthans and Youssef, 2017) and positive organizational scholarship (Cameron et al., 2003) have emerged as influential frameworks for inspiring human change by shifting the focus from deficits and problems to strengths and potential. Within this context, the application of Psychological Capital (PsyCap) has gained traction as a research-based approach to human resource development and performance enhancement in the workplace (Luthans et al., 2007a,b). PsyCap refers to an individual’s psychological resource capacity—a set of positive, state-like traits that can be measured, developed, and managed to improve performance and well-being. In the following section, we provide a brief overview of the four core components of PsyCap: *Hope, Efficacy, Resilience*, and *Optimism* (Luthans and Youssef, 2017).

*Hope* can be understood as a positive psychological state, in contrast to negative emotional states such as hopelessness, despair, or cynicism. In positive psychology, Hope is conceptualized as comprising two key dimensions: willpower and pathways (Snyder, 2002). *Willpower* refers to the motivation and expectancy individuals hold toward achieving a desired goal, while *pathways* represent the cognitive resources that enable individuals to identify multiple strategies for reaching that goal. These alternative pathways help individuals persist in goal pursuit, even when faced with obstacles. Research suggests that positive emotions, such as hope, are associated with pro-environmental behavior (Feldman and Hart, 2018). Specifically, hope appears to foster greater engagement in environmental action, particularly when it involves beliefs in the potential for collective climate change mitigation. This is supported by findings indicating a modest positive correlation (*r* = 0.18) between hope and pro-environmental behavior (Brosch, 2021; Geiger et al., 2021).

*Efficacy* refers to an individual’s belief in their ability to successfully perform specific tasks (Bandura, 1997). While it is considered a universal psychological state, its expression varies across different contexts (Bandura, 1997). Closely related is the concept of collective efficacy, which reflects a group’s shared belief in its capacity to achieve desired outcomes through coordinated action (Bandura, 2000). Although previous research suggests that collective efficacy is a stronger predictor of pro-environmental behavior than self-efficacy (Chen, 2015), emerging evidence indicates that collective efficacy may enhance environmental intentions by strengthening individual self-efficacy (Jugert et al., 2016). This interplay highlights the crucial role of self-efficacy in mobilizing climate action, as individuals who feel confident in their ability to contribute are more likely to engage in environmentally responsible behavior.

*Resilience* is commonly defined as the capacity to recover from adverse or stressful situations (Masten et al., 1990). More broadly, it encompasses the ability of individuals, communities, and systems to survive, adapt, grow, and even transform in response to stressors and shocks when conditions demand it (Ford et al., 2020). In the context of climate change, resilience—both at the individual and ecological levels—represents a set of adaptive processes essential for confronting both immediate and long-term risks. At the individual level, resilience often manifests through emotion regulation strategies, which are critical intrapersonal competencies for sustainability change agents navigating the setbacks and frustrations inherent in climate policy and action (Ayers et al., 2023; Kovacs et al., 2024). These psychological resources enable individuals to maintain engagement and effectiveness in the face of uncertainty and resistance, making resilience a vital component of climate change mitigation efforts.

*Optimism* is characterized by a positive explanatory style, where individuals tend to internalize positive events and externalize negative ones (Peterson, 2000). As a psychological trait, optimism is generally associated with beneficial outcomes, including enhanced well-being and motivation. However, it can also have potential drawbacks, particularly when it leads to unrealistic expectations, disappointment, or self-deception (Peterson, 2000). Optimism may be influenced by self-serving biases, such as *comparative optimism*—the belief that positive events are more likely, and negative events less likely, to happen to oneself than to others. A more problematic form, *unrealistic optimism*, involves the erroneous expectation of positive outcomes, often linked to information-processing biases and maladaptive coping strategies (Radcliffe and Klein, 2002). In the environmental domain, comparative optimism may lead individuals to believe they are less vulnerable to environmental risks than others, potentially undermining their motivation to act (Gifford et al., 2009). While optimism can be psychologically beneficial, it may also have counterproductive implications for environmental policy and risk management by discouraging proactive environmental strategies and fostering complacency.

The Psychological Capital (PsyCap) components—Hope, Efficacy, Optimism, and Resilience—are grounded in robust theory, supported by empirical research, and assessed through validated measurement tools (Luthans et al., 2007a,b; Luthans and Youssef, 2017). Importantly, these components are considered state-like, meaning they are open to development through targeted interventions and learning processes. In work-life settings the higher-order construct of PsyCap has been linked to a wide range of desirable work-related outcomes, including enhanced attitudes, behaviors, and interpersonal trust (Nolzen, 2018; Nguyen et al., 2024). One of the most appealing aspects of PsyCap is its developmental potential, which has inspired a growing body of research on PsyCap interventions. This literature includes both micro-interventions—brief, focused activities—and comprehensive development programs aimed at cultivating psychological resources in organizational settings (Salanova and Ortega-Maldonado, 2019).

The aim of this pilot study is to explore how Psychological Capital (PsyCap)—comprising Hope, Efficacy, Resilience, and Optimism—may contribute to explain individual differences in environmental action. To this end, we conducted a two-part study involving an interdisciplinary group of university students participating in a four-month sustainability summer course on a sail ship. In the first phase, participants completed an individual survey that included the *Psychological Capital Questionnaire* and the *Environmental Activities and Actions Scale*. In the second phase, the same group participated in focus group discussions examining how the four PsyCap components might serve as motivational drivers for engaging in environmental actions.

## Method

### Design and procedure

The two-part study was conducted during the One Ocean Expedition from May to August in 2022, when the University of Bergen hosted an interdisciplinary summer course aboard the sailing vessel Statsraad Lehmkuhl (Eid et al., 2024). The course, *SDG 200 Ocean–Climate–Society*, is a 30 ECTS elective offered to both undergraduate and graduate students, with a particular emphasis on Sustainable Development Goals (SDGs): SDG 14 (Life Below Water), SDG 17 (Partnerships for the Goals), and SDG 13 (Climate Action). The SDG 200 topic was designed as research and teaching expedition and all students gave informed consent to take part in research exploring sustainability issues. This also included student assignments that could be used in anonymized form for research purposes. Data protection/GDPR assessment/registration procedures were approved by the Norwegian Agency for Shared Services in Education and Research (SIKT; Ref.nr. 712,106). The student feedback and evaluations were anonymously collected and stored in accordance with the University of Bergen quality assurance of teaching and education.

For further details, see: https://www4.uib.no/en/studies/courses/sdg200. In the first part of the study, students were invited to complete the 18-item Environmental Actions Scale (Alisat and Riemer, 2015) and the short form of the Psychological Capital Questionnaire (PCQ-12: Luthans et al., 2007a,b). The focus group discussions were conducted 7 days later. After reading a brief description of the four Psychological Capital (PsyCap) components—Hope, Efficacy, Resilience, and Optimism—students participated in one of 18 focus groups, each tasked with the following assignment:

“*The concept of Psychological Capital (PsyCap) is generally seen as a desirable human resource capacity and is associated with desired behavioral outcomes such as productivity, creativity, and safety. Still, few have considered the relevance of PsyCap as a human resource capacity in reaching the SDG goals. Please provide a one-page note from the group discussion where you consider the significance of each PsyCap factor regarding one or more of the SDG outcomes. - Do you think any of the PsyCap factors could be more important than the other?”*

Each of the 18 focus groups submitted a written summary (1–2 pages) of their discussions. These reports did not include any identifying information about the students and were subsequently analyzed for research purposes in anonymized form, after all mandatory assignments and formal course evaluations had been completed.

### Sample characteristics

The *SDG 200 Ocean–Climate–Society* course is designed to complement students’ academic backgrounds in fields such as teacher education, biology, social sciences, law, medicine, psychology, anthropology, computer science, design, ecology, and geography (see Eid et al., 2024). Participants were selected from over 400 applicants based on their motivation letters, with the aim of fostering a multidisciplinary and internationally diverse learning environment. The final group included 86 students (24 male and 62 female) from Norwegian and international higher education institutions. The participants represented a diverse range of higher education institutions from Austria, Canada, Fiji, Germany, Greece, Italy, Norway, Peru, and South Africa. The student cohort included individuals of Canadian, Fijian, Finnish, German, Greek, Italian, Norwegian, Papua New Guinean, Peruvian, South African, and Swedish nationalities. As a result, the student group represented a wide range of academic backgrounds and demonstrated strong motivation and commitment to sustainability and environmental action. The first 5 weeks of the four-month course were spent crossing the Pacific Ocean aboard a sailing vessel, traveling from Valparaíso, Chile to Tahiti, French Polynesia. All 86 students participated in the educational activities and contributed to one of the 18 focus group sessions. Of these, 55 students (64%) returned the research survey. One respondent had not completed the PCQ scale leaving 54 valid responses to be included in the quantitative analysis. The survey respondents included 19 males and 36 females, aged 21 to 33 years (*M* = 24.05, SD = 2.73). Participating in the research survey was voluntary and based on informed consent. Completed surveys were not processed until after the voyage was completed. Drop-out had no consequences for their academic standing. The primary reasons for non-participation were fatigue and the need to prioritize sleep, mandatory coursework, and their daily assignments onboard the ship. When comparing the demographics of the respondents to the total sample, 19 out of 24 male (79%) and 36 out of 62 female (58%) students completed the survey.

### Instruments

In the first part of the study, students completed the Environmental Activities and Action Scale (EAAS; Alisat and Riemer, 2015), which assesses the frequency of engagement in civic actions aimed at producing collective environmental impact in the last 6 months. The EAAS consists of 18 items rated on a 5-point Likert scale ranging from 0 (*never*) to 4 (*frequently*), where respondents indicate their level of agreement with statements describing actual (not intentional) environmental behaviors. The scale captures two subdimensions of system-level environmental action: Participatory Actions, such as supporting or contributing to environmental initiatives (e.g., “I have financially supported an environmental cause”), and Leadership Actions, such as organizing events or mobilizing others toward environmental solutions (e.g., “I have organized an environmental protest/rally”). The internal consistency of the total EAAS and the subscales of Participatory Action and Leadership Action as measured by Cronbach’s alpha, was 0.89, 0.87 and 0.66, respectively (*n* = 54). Psychological Capital (PsyCap) was assessed using the abbreviated 12-item version of the original 24-item Psychological Capital Questionnaire (PCQ; Luthans et al., 2007b). This short form includes items representing all four PsyCap components: Hope (4 items—2 assessing agentic capacity and 2 assessing pathways thinking), Efficacy (3 items), Resilience (3 items) and Optimism (2 items). Students rated their agreement with each statement on a 6-point Likert scale ranging from 1 (*Strongly disagree*) to 6 (*Strongly agree*). Previous studies have shown that 12-item scales are well suited to measure the core components of PsyCap (Lorenz et al., 2016) and in the context of a sail ship, a brief measure considered preferable to elicit compliance to take part in the study. The internal consistency of the total PCQ-12, as measured by Cronbach’s alpha, was 0.71 (*n* = 54). Following from Ferketich (1991) corrected item–total correlations between 0.30 and 0.70 are generally considered indicators of satisfactory item discrimination and scale coherence. However, an inspection of the item-total correlations of the PsyCap scale revealed that four items; Hope (1), Resilience (2) and Optimism (1) had Corrected Item–Total Correlation (CITC) scores <0.30 and were excluded from the analysis. An abridged 8-item PsyCap scale with all four components was therefore used in the subsequent analysis. This abridged 8-item scale included Hope (three items), Efficacy (three items), Resiliency (one item) and Optimism (one item) and had a total Cronbach’s alpha of 0.77 (*n* = 54) with CITC scores from 0.31 to 0.61. The Hope and Efficacy components had a Cronbach’s alpha of 0.77 and 0.79, respectively. To assess the robustness of using the abridged scale, we conducted a sensitivity check. The abridged 8-item scale correlated strongly with the full 12-item scale (*r* = 0.93, *p* > 0.001) and showed improved internal consistency compared to the 12-item scale (*α* = 0.71 vs. α = 0.77).

### Statistical analyses and procedure

Descriptive statistics, including means (M) and standard deviations (SD), were calculated to provide an overview of the central tendency and variability for each variable. To assess the relationships between variables, bivariate correlation analyses were conducted. Pearson correlation coefficients were used to quantify the strength and direction of linear associations among the constructs. In addition, 95% confidence intervals were computed for each correlation estimate, providing information about the precision of these estimates and the degree of statistical uncertainty.

To further investigate the unique contribution of each PsyCap component on the environmental action outcomes, three multiple regression analysis were performed. In these models, unstandardized coefficients (B) were estimated to represent the expected change in the outcome variable for a one-unit increase in each predictor, while standardized coefficients (*β*) were included to enable comparison of the relative strength of predictors measured on different scales. Standard errors (SE B) were calculated to quantify the sampling variability associated with the coefficient estimates. *t*-values and corresponding *p*-values were computed to evaluate the statistical significance of each PsyCap component within the model. To assess the precision of the regression estimates, bias-corrected and accelerated (BCa) bootstrap confidence intervals were generated. Variance inflation factors (VIF) were also computed to evaluate multicollinearity among the PsyCap components, ensuring that the estimated effects were not distorted by high intercorrelations. Together, these regression procedures offer a comprehensive examination of the extent to which each predictor contributes uniquely to the outcome variables within the broader analytical framework of the study.

### Analysis of the focus group reports

A directed content analysis (DCA) approach was used to examine how the four components of Psychological Capital (PsyCap)—Hope, efficacy, resilience, and Optimism—were described in relation to environmental action. DCA was selected because the aim was to analyze the transcripts using an existing theoretical framework, rather than to develop new categories (Hsieh and Shannon, 2005). The four PsyCap components therefore served as predefined coding categories.

The qualitative dataset consisted of 18 short focus-group transcripts (approximately 1–1.5 pages each). Each transcript gave a summary of the focus group assignment (see procedure). The coding procedures and workflow were conducted by two researchers (JE, JT) following a structured process:

*Initial reading:* first, both researchers read all transcripts to gain an overview and to identify where the four PsyCap components were mentioned.
*Deductive coding:* then the researchers noted positive or negative references to Hope, efficacy, resilience, and Optimism. Coding was done manually using printed transcripts and notes.
*Valence coding:* each PsyCap component was categorized as either *positive* (when the component was described as supporting environmental action) or *negative* (when the component was described as weak, absent, or potentially hindering environmental action).
*Interaction coding:* finally, when a transcript described how one PsyCap component influenced or interacted with another (e.g., optimism boosting efficacy), these were noted as positive or negative interactions.

After all transcripts were assessed, the two researchers compared and discussed their notes. They reviewed disagreements, clarified borderline cases, and agreed on a final set of tallies for each PsyCap category. These tallies form the basis of Table 1 presented in the Results. The two researchers then jointly reviewed all transcripts again to identify short quotations that illustrated how each PsyCap component was evaluated. Quotations were selected through discussion to ensure they represented typical reasoning and were clearly connected to the final coding.

**Table 1:** Summary of the 18-focus group (FG) assessments of how PsyCap factors impact environmental action and how they may interact with other PsyCap factors counted once per group mentioning it.

PsyCap	Impact on environmental action		Interacts with other PsyCap factors	
	Negative	Positive	Negative	Positive
Hope		18		2
Resilience		18		4
Efficacy		18		5
Optimism	10	18	7	13

Several measures were used to enhance the study’s analytic trustworthiness. Such measures included coder triangulation, keeping aa consensus approach to disagreement and included a peer debriefing where a third researcher (KE) reviewed the analytical text to ensure coherence, interpretive consistency, and clarity of argumentation. Saturation was considered likely to have been achieved due to the assignment structure and that responses showed substantial thematic repetition. Thus, additional transcripts were unlikely to yield new categories within the predefined DCA framework. The final output of the analysis consisted of an analytical narrative and a summary table (Table 1) showing the valance (positive or negative) for each PsyCap component and its interactions with other components.

## Results

Table 2 presents the descriptive statistics and intercorrelations among all study variables, supplemented with bias-corrected and accelerated (BCa) 95% bootstrapped confidence intervals based on 5,000 resamples. Means and standard deviations indicates expected variability across the environmental action and psychological capital scales. The correlation matrix demonstrates that the three environmental action indices—Participatory Actions, Leadership Actions, and Total EAA—were strongly and positively correlated with each other. Consistent with PsyCap theory, the PsyCap composite score and its subcomponents (Efficacy, Hope, Resilience and Optimism) returned moderate to strong positive correlations, reflecting their shared underlying resource structure.

**Table 2:** Descriptive statistics, intercorrelations, and 95% bootstrapped confidence intervals for participatory actions, leadership actions, EAA total, and the PsyCap components of efficacy, hope, resilience, optimism and PCQ-8 total (*N* = 54).

Variable	M	SD	PA	LA	EAA	EFF	HOPE	RES	OPT	PCQ-8
PA	0.146	0.079	–							
LA	0.029	0.044	0.774*** [0.662, 0.855]	–						
EAA	1.693	1.091	0.979*** [0.970, 0.987]	0.888*** [0.811, 0.933]	–					
EFF	4.383	1.051	0.420*** [0.161, 0.637]	0.386*** [0.184, 0.553]	0.431*** [0.205, 0.621]	–				
HOPE	4.568	0.889	0.216 [ns]	0.293*** [0.042, 0.491]	0.253 [ns]	0.315*** [0.038, 0.562]	–			
RES	4.519	0.966	0.381*** [0.135, 0.584]	0.345*** [0.082, 0.552]	0.389*** [0.150, 0.587]	0.358*** [0.110, 0.557]	0.119 [ns]	–		
OPT	5.241	0.725	0.112 [ns]	0.082 [ns]	0.108 [ns]	0.182 [ns]	0.359*** [0.039, 0.597]	0.142 [ns]	–	
PCQ-8	4.678	0.603	0.449*** [0.218, 0.623]	0.439*** [0.230, 0.595]	0.469*** [0.255, 0.623]	0.750*** [0.629, 0.843]	0.662*** [0.478, 0.794]	0.643*** [0.468, 0.795]	0.569*** [0.294, 0.745]	–

Values below the diagonal represent bootstrapped Pearson correlations (5,000 samples) with BCa 95% confidence intervals. Significance stars are based on two-tailed *p*-values: *p < 0.05. **p < 0.01. ***p < 0.001.

PsyCap efficacy showed small-to-moderate positive correlations with all environmental action indicators, with BCa confidence intervals consistently above zero, indicating stable associations. PsyCap resilience showed a similar pattern, though some intervals overlapped zero, reflecting reduced precision due to sample size. Hope and Optimism demonstrated weaker associations with environmental action variables, and several of their bootstrapped intervals included zero, suggesting that these components may play a less central role in predicting environmental action in this pilot sample. Importantly, all correlations in Table 2 should be interpreted cautiously given the exploratory context and modest sample size.

To examine the extent to which psychological capital components explained environmental action attitudes and behaviors, three multiple regression analyses were conducted using efficacy, Hope, Resilience, and Optimism as independent variables. Dependent variables included the EAA Total scale, the Participatory Actions subscale, and the Leadership Actions subscale. Bootstrapped standard errors and bias-corrected and accelerated (BCa) 95% confidence intervals based on 5,000 samples were computed to enhance robustness.

### EAA total score

The full model in Table 3 significantly explained variance in EAA Total scores, *F*(4, 49) = 4.41, *p* = 0.004, explaining 26.5% of the variance (*R*^2^ = 0.265, Adjusted *R*^2^ = 0.205). Efficacy (*B* = 0.308, *β* = 0.296, *p* = 0.036) and Resilience (*B* = 0.307, β = 0.271, *p* = 0.045) showed meaningful unique contributions explaining 8.6 and 8.0% of the variance, respectively. BCa 95% CIs for both did not include zero, reinforcing their stability. Neither Hope nor Optimism significantly explained EAA Total.

**Table 3:** Multi-panel regression summary: EAA total, participatory actions, leadership actions from PsyCap components (*N* = 54).

Predictor	*B*	SE B	*β*	*t*	*p*	95% BCa CI	VIF
EAA total							
Efficacy	0.308	0.143	0.296	2.156	0.036	[0.030, 0.611]	1.26
Hope	0.171	0.167	0.139	1.022	0.312	[−0.110, 0.473]	1.24
Resilience	0.307	0.149	0.271	2.062	0.045	[0.015, 0.603]	1.16
Optimism	−0.052	0.199	−0.035	−0.263	0.794	[−0.397, 0.277]	1.16
Participatory actions							
Efficacy	0.022	0.011	0.298	2.138	0.038	[−0.001, 0.045]	1.26
Hope	0.009	0.012	0.096	0.697	0.489	[−0.013, 0.031]	1.24
Resilience	0.022	0.011	0.265	1.988	0.052	[−0.001, 0.045]	1.16
Optimism	−0.002	0.015	−0.015	−0.110	0.913	[−0.027, 0.021]	1.16
Leadership actions							
Efficacy	0.010	0.006	0.246	1.754	0.086	[0.001, 0.022]	1.26
Hope	0.011	0.007	0.213	1.534	0.132	[−0.001, 0.023]	1.24
Resilience	0.011	0.006	0.242	1.801	0.078	[−0.002, 0.023]	1.16
Optimism	−0.005	0.008	−0.074	−0.548	0.586	[−0.019, 0.010]	1.16

BCa, bias-corrected and accelerated bootstrap confidence intervals. VIF, Variance Inflation Factor. All coefficients represent unstandardized (B) and standardized (β) regression estimates.

### Participatory actions subscale

A second model explaining participatory actions in Table 3 was also significant, *F*(4, 49) = 3.99, *p* = 0.007, accounting for 24.5% of the variance (*R*^2^ = 0.245, Adjusted *R*^2^ = 0.184). A similar pattern emerged as in the total score: efficacy showed a significant positive effect (*B* = 0.022, β = 0.298, *p* = 0.038; *sr*^2^ = 0.085), and resilience approached significance (*p* = 0.052; *sr*^2^ = 0.075). Bootstrap BCa intervals for both efficacy and resilience marginally touched zero but were largely consistent with the classical CIs. Hope and Optimism showed small, non-significant contributions.

### Leadership actions subscale

The third model in Table 3, explaining leadership-oriented environmental actions, was also significant, *F*(4, 49) = 3.76, *p* = 0.010, explaining 23.5% of the variance (*R*^2^ = 0.235, Adjusted *R*^2^ = 0.173). Although no PsyCap components reached conventional significance, Efficacy, Resilience, and Hope all demonstrated modest unique effects (*sr*^2^ = 0.060–0.063), and their bootstrap BCa confidence intervals approached but did not fully exclude zero. The general pattern mirrors the other two outcomes: psychological capital components—particularly Efficacy and Resilience—maintain small but consistent contributions.

Across all three models, multicollinearity was minimal, with VIF values between 1.15 and 1.26 and tolerance values well above 0.70. The stability of the PsyCap components contributions across bootstrap CIs suggests that the relationships, while modest, are consistent across the EAA constructs. Taken together, the results from the quantitative study suggest that efficacy and resilience are the most consistently influential components of psychological capital in explaining variance in environmental attitudes, participatory behaviors, and leadership-oriented action. The bootstrapped estimates bolster confidence in the robustness of these effects. Hope and Optimism, while theoretically aligned with positive psychological functioning, showed minimal unique explanatory value across the three EAA dimensions.

#### Focus group assessments of the PsyCap factors

All 18 focus groups (FG 1–18) engaged in discussions covering each of the four PsyCap components. Findings are presented in line with the directed content analysis, organized by the four Psychological Capital (PsyCap) components and their valence, followed by interactions among components. The groups explored both the positive and negative implications of PsyCap in relation to environmental action and the Sustainable Development Goals (SDGs), as well as the potential cumulative effects of combining PsyCap factors. While many groups found it challenging to identify a single PsyCap factor as most critical for environmental action, there was broad consensus that Hope, Efficacy, and Resilience were particularly important and generally perceived as having a positive influence. Optimism, however, emerged as a more nuanced and occasionally controversial factor. Although all groups acknowledged its potential benefits, ten groups also highlighted possible drawbacks of excessive optimism, such as underestimating the urgency or complexity of environmental challenges. A key observation from the discussions was the perception of Hope, Resilience, and Efficacy as “force multipliers”—factors that, when combined with one another, could amplify their overall impact on environmental action. Patterns are described as indicative of how participants framed environmental action within the assignment context and are not claimed as exhaustive. A summary of mentions and interactions by category appears in Table 1.

##### Optimism

Across the focus groups, Optimism was generally described as beneficial in motivating for environmental action. Although optimism could help sustain momentum when working toward long-term goals excessive optimism could present a potential downside. As one of the groups emphasized, *“However, too optimistic goals can seem impossible to accomplish and therefore undermine the purpose of creating optimism, but rather make the targets seem impossible to reach”* (FG-1; Optimism-negative). At the same time, many participants highlighted the constructive role optimism can play when viewed as part of a broader set of PsyCap resources. One group articulated this integrative perspective clearly: *“Optimism is important for the success of the SDGs because lack of optimism reduces our Hope, Efficacy, and Resilience. We need as much Hope, Efficacy, and Resilience as possible to conquer our SDGs, and Optimism can help us achieve this.”* (FG-17; Optimism—positive interaction). However, not all groups agreed that Optimism consistently supports environmental action. A few warned that excessive or uncritical optimism may inadvertently weaken other PsyCap components over time, for instance, by reducing preparedness for setbacks or minimizing the need for practical planning. As one group expressed: *“Overly optimistic mindsets can lead to long-term reduction of other factors such as Resilience and Hope.”* (FG-13; Optimism—negative interaction). Taken together, these perspectives illustrate that Optimism was not uniformly viewed in the same way across the dataset. Overall, the accounts portray Optimism as a potentially helpful but context-dependent PsyCap resource—one that may support environmental action when balanced with realistic assessment and aligned with Hope, Efficacy, and Resilience.

##### Hope

Across the focus groups, participants frequently reflected on the conceptual relationship between Hope and Optimism, noting that the two constructs can be difficult to distinguish. This ambiguity appeared in several transcripts, where participants described overlap in everyday usage. As one group explained: *“Regarding the terms of Optimism and Hope, we struggled to differentiate the two, but this might be because they overlap a lot in their common (layperson) meaning.”* (FG-5; Hope–Optimism conceptual link). This ambiguity was a recurring theme across discussions. Other groups offered more nuanced interpretations by attempting to distinguish the constructs temporally. *“[…] Optimism is needed to achieve hope and exists more in the present to allow us to achieve hope which exists in the future.”* (FG-8; Hope–Optimism positive interaction). This perspective positions Optimism as a precursor or enabling condition for hope, which is oriented toward future outcomes and sustained motivation. Beyond conceptual reflections, several groups emphasized the foundational role of hope in the broader sustainability agenda. As one group stated: *“Hope is the base of these goals and ties all together, as without it there would never be a vision similar to that outlined in [SDG] goal 17 and the agenda as a whole.”* (FG-4; Hope—positive). This view positions Hope not only as a motivational resource but also as a strategic driver linking long-term visions to specific SDG targets. Interestingly, several focus groups suggested that different PsyCap factors may play distinct roles at various stages of the sustainability process. Hope and Optimism were seen as particularly important during the initial phases of SDG formulation and vision-building, while resilience and Efficacy were considered more critical for the implementation and execution of environmental actions. Although several groups mentioned this temporal differentiation, such patterns should be interpreted cautiously, as references reflect the presence of ideas rather than their relative weight or frequency within the dataset.

##### Resilience and efficacy

Several focus groups discussed *Resilience* and *Efficacy* as closely interconnected, like the relationship observed between *Hope* and *Optimism*. Several groups framed Resilience and Efficacy as mutually reinforcing capacities that help translate long-term sustainability visions into concrete environmental actions. One group emphasized both their importance and the perceived gap between these qualities and real-world leadership: *“After discussing in our group, we concluded that Efficacy and Resilience may be the most important factors if we are to achieve the [SDG] goals, but ironically, those [factors] are the scarcest to find in the stakeholders [politicians and governments] who hold the power.”* (FG-2; Efficacy–Resilience—negative observation). Other groups highlighted the difficulty of ranking these two PsyCap components, underscoring their interdependence. As one group put it: *“Between Resiliency and Efficacy we could not rank them in terms of importance. We concluded that they both support each other.”* (FG-5; Efficacy–Resilience positive interaction). This sentiment was echoed by FG-4, which underscored the practical and action-oriented role of these two components: *“When it comes to Resilience and Efficacy, we have argued that they play an integral role in actually implementing policy and measures to achieve the goals that have been inspired by our collective Hope and Optimism.”* (FG-4; Efficacy–Resilience—positive). Together these reflections suggest a pattern in which Hope and *Optimism* were perceived as important during early stages of sustainability work—such as formulation visions and articulating goals—while *Resilience* and *Efficacy* were viewed as critically important for translating those visions into concrete environmental actions. However, these tendencies should be interpreted with caution. The number of comments referring to Resilience and Efficacy reflects how often the topics arose, not necessarily their relative importance across the dataset. The focus group discussions also produced perspectives that emphasized how all four PsyCap factors were important and served valued purposes, although in different ways, likening them to everyday needs: *“The four factors represent the four legs of a stool, and all of them are needed to keep it in balance”* (FG-14; All components—integrative view). Another group had arrived at the conclusion that all the PsyCap factors served a purpose in addressing complex problems such as environmental action: *“Increasing the feelings of Hope, Efficacy, Resilience, and Optimism in a group will result in a higher ability to cope with problems”* (FG-16; All components—positive). Taken together, these accounts portray Resilience and Efficacy as the PsyCap components most closely associated with sustaining effort, managing obstacles, and ensuring follow-through in environmental action—yet also highlight that students often saw the four PsyCap dimensions as complementary rather than competing, each contributing to sustainability action in distinct but interconnected ways.

## Discussion

While research on positive organizational behavior has significantly advanced our understanding of human motivation, productivity, and workplace dynamics (Peterson et al., 2011; Stajkovic and Luthans, 1998), its potential contribution to sustainability efforts remains largely underexplored. To begin addressing this gap, this pilot study offers an exploratory examination of whether—and in what ways—the Psychological Capital (PsyCap) components of Hope, Efficacy, Resilience, and Optimism may be relevant for supporting environmental action within this specific context, with all findings interpreted considering the study’s limited scope and boundary conditions.

The quantitative analysis suggests that PsyCap Efficacy and Resilience have the strongest unique associations with EAA total scores, whereas Hope and Optimism did not contribute additional explanatory power once the shared variance among the PsyCap components was accounted for. These results offer a preliminary indication that facets related to confidence in one’s abilities (Efficacy) and capacity to recover and adapt (Resilience), may be particularly relevant for understanding environmental action in this sample.

Given evidence that behavioral spillovers are limited (Maki et al., 2019), interventions that build Resilience and Efficacy could help maintain consistency across multiple behaviors. The classification by Van Valkengoed et al. (2022) suggests PsyCap can be integrated as a motivational determinant in intervention design, complementing normative and informational strategies. Meta-analytic findings on identity (Udall et al., 2021) indicate strong links to PEB. PsyCap may interact with identity by enabling individuals to translate group norms into sustained action, reinforcing collective efficacy.

In the present study, the PsyCap components of Efficacy and Resilience were positively associated with environmental action, suggesting that these factors play a critical role in mobilizing climate-related engagement. This finding aligns with previous research by Chen (2015), who demonstrated that collective efficacy beliefs predict pro-environmental behavior, and Jugert et al. (2016), who found that collective efficacy enhances pro-environmental intentions by strengthening individual self-efficacy. A high-engagement educational context such as the sailing expedition may amplify the role of Efficacy and Resilience because it provides mastery experiences, feedback, and opportunities to navigate realistic environmental challenges. These conditions strengthen students’ beliefs in their ability to act and their capacity to persist—two factors consistently linked to action intentions and sustained behavior. This dynamic is theoretically interesting because it highlights how PsyCap components may become more influential under conditions of active participation, providing a context-sensitive extension of both Social Cognitive Theory (Bandura, 1977) and PsyCap theory. Empirical studies in sustainability education (Zhang and Cao, 2025) and educational psychology (Cassidy, 2015; Qamar and Akhter, 2020) support the idea that high-engagement settings enhance both self-efficacy and resilience, thereby increasing readiness for climate- and sustainability-related action. These insights point to a valuable leadership strategy: fostering shared beliefs in the capacity to achieve desired environmental outcomes through collective efficacy. Atshan et al. (2020) caution that the relationship between environmental awareness, concern, attitude, and behavior is complex and often indirect, which may help explain the slow progress toward achieving the SDGs. In this context, the association between Resilience and environmental action observed in our study is particularly relevant. It supports the notion that resilience is essential for navigating setbacks, managing frustration, and developing emotional regulation strategies—skills that are vital for sustainability change agents working to advance climate policies (Ayers et al., 2023; Kovacs et al., 2024). Accordingly, a viable leadership approach may involve preparing individuals and communities to cope with grief and disappointment as part of the long-term sustainability journey.

An overarching observation across both parts of the study is the strong alignment between the empirical findings from the survey and the qualitative insights from the focus group discussions. Notably, the relationship between Resilience and Efficacy was consistently emphasized. As one group noted, *“Between Resiliency and Efficacy we couldn’t rank them in terms of importance. We concluded that they both support each other”* (FG-5). This sentiment reflects the broader consensus among students: Efficacy was seen as essential for problem-solving and task completion, while Resilience was viewed as critical for maintaining persistence and recovering from setbacks in the face of adversity and resistance. Together, these factors were perceived as mutually reinforcing and vital for sustained environmental action and progress toward the SDGs.

Park et al. (2020) describe hope as a complex and multifaceted emotional state, noting that its influence on environmental action depends on various factors, including goal orientation, perceived feasibility, societal and personal norms, personality traits, and group identity. In the present study, hope was conceptualized in line with Snyder’s (2002) framework, which defines it as comprising both *willpower* and *pathways thinking*. These components are essential for leadership actions aimed at fostering pro-environmental behavior (Feldman and Hart, 2018; Geiger et al., 2021).

This conceptualization aligns with our findings, where hope was significantly correlated with the EAAS subdimension of *Leadership action*, underscoring the importance of identifying viable pathways toward desired environmental outcomes. A notable insight from the study is that Resilience, Efficacy, and Hope were perceived by students as “force multipliers”—factors that, when combined, enhance the overall impact of PsyCap on environmental action. This suggests that effective leadership should not only promote goal clarity and strategic pathways but also cultivate emotional resilience and practical efficacy to sustain motivation in the face of setbacks. As Park et al. (2020) notes, grief and hopelessness are often part of the emotional labor involved in conservation and environmental work. Therefore, leadership that actively fosters hope and provides a compelling vision for the future may play a crucial role in sustaining long-term pro-environmental behavior.

In the present study, the PsyCap factor of Optimism was not significantly correlated with environmental action. While most focus groups viewed Optimism as a generally positive asset, many also highlighted potential risks associated with an overly optimistic mindset. As one group noted, *“Too optimistic goals can seem impossible to accomplish and therefore undermine the purpose of creating optimism”* (FG-1). This concern is supported by research in cognitive psychology, which has shown that unrealistic optimism can lead to information-processing biases such as selective perception, avoidant coping, and procrastination (Radcliffe and Klein, 2002).

Although Optimism did not significantly predict environmental action intentions in this study, this null finding is itself theoretically informative. In traditional organizational settings, Optimism is typically associated with enhanced motivation and goal pursuit. However, in environmental contexts—where challenges are collective, long-term, and often perceived as beyond individual control—forms of comparative or unrealistic Optimism may dampen the sense of personal responsibility or urgency needed for action. Research on environmental risk perception shows that individuals often exhibit comparative optimism, believing they are less personally vulnerable to environmental hazards than others, a pattern that does *not* translate into greater pro-environmental behavior (Pahl et al., 2005). Furthermore, comparative optimism— the belief that one is less likely than others to experience negative outcomes—may foster complacency in the environmental domain. For example, assumptions like *“sea level rise will not affect my way of life”* can diminish personal engagement and urgency (Gifford et al., 2009). Several focus groups expressed skepticism about the implications of unrealistic optimism for environmental policy and risk management. FG-13 warned that *“Overly optimistic mindsets can lead to long-term reduction of other factors such as Resilience and Hope.”* These findings suggest that maintaining realistic optimism, while recognizing both progress and challenges, is a crucial aspect of fostering sustained environmental action. Similarly, research on environmental health risks finds that comparative optimism can lead to reduced protective action, as individuals underestimate their own exposure and behavioral responsibility (Wolde et al., 2019). The absence of a direct optimism effect therefore suggests that optimism may operate differently in sustainability domains than in workplace performance, pointing to an important boundary condition in PsyCap theory. This divergence highlights the need to distinguish between *constructive optimism* that energizes action and *complacent optimism* that reduces perceived agency, opening a novel line of inquiry in the application of PsyCap to environmental action. Although, the results from this exploratory study should be interpreted carefully, the null finding that Optimism is not related to environmental action may provide an early empirical signal that environmental contexts may require a revised or extended understanding of PsyCap—especially when Optimism interacts negatively with Efficacy or Resilience. If Optimism reduces perceived personal or collective risk (“things will work out anyway”), it may weaken individual action intentions even though it generally helps in workplace settings. Unlike work outcomes, environmental outcomes rarely depend on individual effort alone. Optimism may therefore detach motivation rather than reinforce it. Taken together, these findings suggest that Optimism may be a *conditional* rather than a universally beneficial resource in sustainability contexts, underscoring the importance of expanding PsyCap theory in future research to account for domain-specific forms of motivation, risk perception, and agency.

Some focus groups emphasized that different PsyCap factors may play distinct roles at various stages of the long-term process of addressing sustainability challenges. This process-oriented perspective offers valuable insight into the dynamic nature of PsyCap, particularly given that environmental action often unfolds over extended periods. Several groups suggested that Hope and Optimism are especially important during the initial stages—when ambitions are formulated and pathways toward the SDGs are mapped. These factors were seen as essential for inspiring vision, generating momentum, and fostering belief in the possibility of change.

In contrast, Resilience and Efficacy were considered more critical in the later stages of the process, where practical problem-solving, persistence, and the ability to recover from setbacks become central to achieving environmental outcomes. This staged view of PsyCap highlights the importance of tailoring leadership and educational strategies to support different psychological capacities at different phases of sustainability work.

### Limitations

Several limitations of this exploratory study should be acknowledged. First, the study relied on a modest sample size (*N* = 54), which increases the risk of both Type I and Type II errors and limits the stability of regression coefficients. Second, because the PsyCap components were moderately intercorrelated, the unique effects observed here may be sensitive to measurement error or sampling fluctuation. Third, the cross-sectional design precludes any causal inference; it is equally plausible that EAA action enhances Efficacy and Resilience, or that all three are shaped by third variables such as prior training, leadership experiences, or personality. Although the abridged 8-item PsyCap scale appeared to have satisfactory sensitivity, it would be recommended to use the full 24-item scale in future studies. Finally, the PsyCap subscales represent related facets of a broader construct, and partialling them out in a regression model can sometimes obscure theoretically meaningful shared variance. Despite these limitations, the findings provide useful starting points for future research. Subsequent studies with larger and more diverse samples could test whether Efficacy and Resilience consistently emerge as key contributors to EAA outcomes and whether these results replicate using longitudinal or experimental designs. Future work should include more diverse participant groups and incorporate independent or observational measures to enhance reliability and reduce potential bias. Additional research might also examine PsyCap along with potential mediators (e.g., collective efficacy/social identity) and moderators (e.g., organizational climate, prior training) that help explain why certain PsyCap components relate more strongly to EAA. Finally, the sample consisted of selected individuals with a pre-existing interest in environmental and sustainability issues. While this limits generalizability, it could also be considered a strength in the focus group context: participants’ engagement and firsthand experience enabled them to identify nuanced differences in how PsyCap components interact and contribute to environmental action. The study thus illustrates how qualitative approaches or multi-method assessments could enrich future understanding of how individuals draw on psychological resources when engaging in EAA-relevant tasks.

### Contributions and implications

This two-part exploratory study offers initial insights into the potential role of Psychological Capital (PsyCap)—comprising Hope, Efficacy, Resilience, and Optimism—in shaping environmental action. By examining PsyCap as a higher-order construct, the study adds preliminary empirical support for the idea that psychological resources like Efficacy and Resilience, may be relevant to an environmental action and advocacy context. Given the state-like nature of PsyCap, these findings also point to promising directions for future research, particularly regarding whether PsyCap components may mediate or moderate environmentally responsible behavior in more rigorous, longitudinal, or intervention-based designs.

The study further highlights the value of investigating PsyCap as a dynamic process rather than a static trait. PsyCap components may function differently over time, and they may interact with individual and contextual factors that influence environmental citizenship. While Hope and Optimism may help individuals initiate engagement and maintain a long-term orientation, Efficacy and Resilience may be more pertinent to navigating obstacles, problem solving, and sustaining involvement. Although these patterns cannot be generalized beyond the study context, they suggest the potential importance of adopting a process-focused approach to leadership and education in sustainability—one that recognizes that different psychological resources may matter at different stages of environmental action.

These insights also raise the possibility that PsyCap components could operate as pathways for strengthening social capital and supporting forms of environmental citizenship that are collaborative, voluntary, and oriented toward long-term sustainability. Based on these preliminary indications, the study tentatively proposes that leaders seeking to encourage and sustain environmental action may benefit from strategies such as:

1. Promoting hope by communicating a credible and motivating vision of possible ways forward.
2. Fostering realistic optimism by acknowledging progress while remaining transparent about challenges.
3. Strengthening collective efficacy by reinforcing shared beliefs in the group’s ability to contribute to desired outcomes.
4. Preparing for setbacks by encouraging resilient responses to frustration, grief, or disappointment.

Together, these considerations illustrate how PsyCap may play a multifaceted role in supporting sustainability efforts, while also underscoring the need for more theory-driven and longitudinal research to clarify underlying mechanisms and assess the potential impact of strengthening specific PsyCap components on sustained environmental action.

## Data Availability

The original contributions presented in the study are included in the article/Supplementary material, further inquiries can be directed to the corresponding author.

## References

[ref1] Alisat S. Riemer M. (2015). The environmental action scale: development and psychometric evaluation. J. Environ. Psychol. 43, 13–23. doi: 10.1016/j.jenvp.2015.05.006

[ref2] Atshan S. Bixler R. P. Rai V. Springer D. W. (2020). Pathways to urban sustainability through individual behaviors: the role of social capital. Environ. Sci. Pol. 112, 330–339. doi: 10.1016/j.envsci.2020.07.005

[ref3] Ayers J. Missimer M. Bryant J. (2023). Intrapersonal capacities for sustainability: a change agent perspective on the ‘inner dimension’ of sustainability work. Sustain. Sci. 18, 1181–1197. doi: 10.1007/s11625-022-01288-8

[ref4] Bandura A. (1977). Self-efficacy: toward a unifying theory of behavioral change. Psychol. Rev. 84, 191–215. doi: 10.1037/0033-295X.84.2.191 847061

[ref001] Bandura A. (1997). Self-Efficacy: The Exercise of Control. New York, NY: W. H. Freeman.

[ref5] Bandura A. (2000). Exercise of human agency through collective efficacy. Curr. Dir. Psychol. Sci. 9, 75–78. doi: 10.1111/1467-8721.00064

[ref6] Barth M. Masson T. Fritsche I. Fielding K. Smith J. R. (2021). Collective responses to global challenges: the social psychology of pro-environmental action. J. Environ. Psychol. 74:101562. doi: 10.1016/j.jenvp.2021.101562

[ref7] Brosch T. (2021). Affect and emotions as drivers of climate change perception and action: a review. Curr. Opin. Behav. Sci. 42, 15–21. doi: 10.1016/j.cobeha.2021.02.001

[ref8] Brundiers K. Barth M. Cebrián G. Cohen M. Diaz L. Doucette-Remington S. . (2021). Key competencies in sustainability in higher education—toward an agreed-upon reference framework. Sustain. Sci. 16, 13–29. doi: 10.1007/s11625-020-00838-2

[ref002] Cameron K. S. Dutton J. E. Quinn R. E. (2003). Positive Organizational Scholarship: Foundations of a New Discipline. Berrett-Koehler Publishers.

[ref9] Cassidy S. (2015). Resilience building in students: the role of academic self-efficacy. Front. Psychol. 6:1781. doi: 10.3389/fpsyg.2015.01781, 26640447 PMC4661232

[ref10] Chen M. F. (2015). Self-efficacy or collective efficacy within the cognitive theory of stress model: which more effectively explains people's self-reported pro-environmental behavior? J. Environ. Psychol. 42, 66–75. doi: 10.1016/j.jenvp.2015.02.002

[ref004] Dietz T. Gardner G. T. Gilligan J. Stern P. C. Vandenbergh M. P. (2009). Household actions can provide a behavioral wedge to rapidly reduce U.S. carbon emissions. Proc. Natl. Acad. Sci. 106, 18452–18456. doi: 10.1073/pnas.0908738106 19858494 PMC2767367

[ref11] Eid J. Aanerud M. Enberg K. (2024). Teaching sustainability at the high sea: the “one ocean expedition”. Sustain. Sci. 19, 347–359. doi: 10.1007/s11625-023-01419-9

[ref12] Feldman L. Hart P. S. (2018). Is there any hope? How climate change news imagery and text influence audience emotions and support for climate mitigation policies. Risk Anal. 38, 585–602. doi: 10.1111/risa.12868, 28767136

[ref13] Ferketich S. (1991). Focus on psychometrics: aspects of item analysis. Res. Nurs. Health 14, 165–168. doi: 10.1002/nur.4770140211, 2047538

[ref14] Fielding K. S. Hornsey M. J. (2016). A social identity analysis of climate change and environmental attitudes and behaviors: insights and opportunities. Front. Psychol. 7:121. doi: 10.3389/fpsyg.2016.00121, 26903924 PMC4749709

[ref15] Ford J. D. King N. Galappaththi E. K. Pearce T. McDowell G. Harper S. L. (2020). The resilience of indigenous peoples to environmental change. One Earth 2, 532–543. doi: 10.1016/j.oneear.2020.05.014

[ref16] Fritsche I. Barth M. Jugert P. Masson T. Reese G. (2018). A social identity model of pro-environmental action (SIMPEA). Psychol. Rev. 125, 245–269. doi: 10.1037/rev0000090, 29265852

[ref17] Fritsche I. Masson T. (2021). Collective climate action: when do people turn into collective environmental agents? Curr. Opin. Psychol. 42, 114–119. doi: 10.1016/j.copsyc.2021.05.001, 34130199

[ref18] Geiger N. Swim J. K. Gasper K. Fraser J. Flinner K. (2021). How do I feel when I think about taking action? Hope and boredom, not anxiety and helplessness, predict intentions to take climate action. J. Environ. Psychol. 76:101649. doi: 10.1016/j.jenvp.2021.101649

[ref19] Gifford R. Scannell L. Kormos C. Smolova L. Biel A. Boncu S. . (2009). Temporal pessimism and spatial optimism in environmental assessments: an 18-nation study. J. Environ. Psychol. 29, 1–12. doi: 10.1016/j.jenvp.2008.06.001

[ref23] Hsieh H.-F. Shannon S. E. (2005). Three approaches to qualitative content analysis. Qual. Health Res. 15, 1277–1288. doi: 10.1177/1049732305276687, 16204405

[ref003] Intergovernmental Panel on Climate Change. (2018). Global Warming of 1.5°C: An IPCC Special Report. Available online at: https://www.ipcc.ch/sr15/

[ref24] Jugert P. Greenaway K. H. Barth M. Büchner R. Eisentraut S. Fritsche I. (2016). Collective efficacy increases pro-environmental intentions through increasing self-efficacy. J. Environ. Psychol. 48, 12–23. doi: 10.1016/j.jenvp.2016.08.003

[ref25] Kovacs L. N. Jordan G. Berglund F. Holden B. Niehoff E. Pohl F. . (2024). Acting as we feel: which emotional responses to the climate crisis motivate climate action. J. Environ. Psychol. 96:102327. doi: 10.1016/j.jenvp.2024.102327

[ref26] Lorenz T. Beer C. Pütz J. Heinitz K. (2016). Measuring psychological capital: construction and validation of the compound PsyCap scale (CPC-12). PLoS One 11:e0152892. doi: 10.1371/journal.pone.0152892, 27035437 PMC4817957

[ref005] Luthans F. (2002). Positive organizational behavior: developing and managing psychological strengths. Acad. Manag. Exec. 16, 57–75. doi: 10.5465/AME.2002.6640181

[ref006] Luthans F. Avey J. B. Avolio B. J. Norman S. M. Combs G. M. (2006). Psychological capital development: toward a micro-intervention. J. Organ. Behav. 27, 387–393. doi: 10.1002/job.373

[ref27] Luthans F. Avolio B. J. Avey J. B. Norman S. M. (2007a). Positive psychological capital: measurement and relationship with performance and satisfaction. Pers. Psychol. 60, 541–572. doi: 10.1111/j.1744-6570.2007.00083.x

[ref28] Luthans F. Youssef C. M. (2017). Psychological capital: an evidence-based positive approach. Annu. Rev. Organ. Psychol. Organ. Behav. 4, 339–366. doi: 10.1146/annurev-orgpsych-032516-113324

[ref29] Luthans F. Youssef C. M. Avolio B. J. (2007b). Psychological Capital. Developing the human competitive edge. New York: Oxford University Press.

[ref30] Macias T. Williams K. (2016). Know your neighbors, save the planet: social capital and the widening wedge of pro-environmental outcomes. Environ. Behav. 48, 391–420. doi: 10.1177/0013916514540458

[ref31] Maki A. Carrico A. R. Raimi K. T. Truelove H. B. Araujo B. Yeung K. L. (2019). Meta-analysis of pro-environmental behavioral spillover. Nat. Sustain. 2, 307–315. doi: 10.1038/s41893-019-0263-9

[ref32] Masten A. S. Best K. M. Garmezy N. (1990). Resilience and development: contributions from the study of children who overcome adversity. Dev. Psychopathol. 2, 425–444. doi: 10.1017/S0954579400005812

[ref33] Microsoft (2024) Microsoft copilot [generative AI]. Available online at: https://copilot.microsoft.com (Accessed March 11, 2026).

[ref34] National Committee for Research Ethics in the Social Sciences and the Humanities (2021/2023). Guidelines for Research Ethics in the Social Sciences and the Humanities. 5th Edn. Oslo: Norwegian National Research Ethics Committees. Available at: https://www.forskningsetikk.no/en/guidelines/social-sciences-and-humanities/guidelines-for-research-ethics-in-the-social-sciences-and-the-humanities/ (Accessed March 11, 2026).

[ref35] Nguyen T. D. Cao T. H. Nguyen T. M. Nguyen T. T. (2024). Psychological capital: a literature review and research trends. Asian J. Econ. Bank. 8, 412–429. doi: 10.1108/AJEB-08-2023-0076

[ref36] Nolzen N. (2018). The concept of psychological capital: a comprehensive review. Manag. Rev. Q. 68, 237–277. doi: 10.1007/s11301-018-0138-6

[ref37] Pahl S. Harris P. R. Todd H. A. Rutter D. R. (2005). Comparative optimism for environmental risks. J. Environ. Psychol. 25, 1–11. doi: 10.1016/j.jenvp.2004.12.004

[ref38] Park A. Williams E. Zurba M. (2020). Understanding hope and what it means for the future of conservation. Biol. Conserv. 244:108507. doi: 10.1016/j.biocon.2020.108507

[ref0001] Peterson C. (2000). The future of optimism. American Psychologist. 55, 44–55. doi: 10.1037/0003-066X.55.1.44 11392864

[ref39] Peterson S. J. Luthans F. Avolio B. J. Walumbwa F. O. Zhang Z. (2011). Psychological capital and employee performance: a latent growth modeling approach. Pers. Psychol. 64, 427–450. doi: 10.1111/j.1744-6570.2011.01215.x

[ref40] Qamar S. Akhter M. (2020). Relationship between students’ self-efficacy and resilience at secondary school level. Bull. Educ. Res., 42, 215–224. Available online at: https://eric.ed.gov/?id=EJ1382173. (Accessed March 11, 2026).

[ref41] Radcliffe N. M. Klein W. M. P. (2002). Dispositional, unrealistic, and comparative optimism: differential relations with the knowledge of risk information and beliefs about personal risk. Personal. Soc. Psychol. Bull. 28, 836–846. doi: 10.1177/0146167202289012

[ref42] Salanova M. Ortega-Maldonado A. (2019) Psychological capital development in organizations: an integrative review of evidence-based intervention programs Zyl L. E. Van Rothmann S. Positive Psychological Intervention Design and Protocols for Multi-Cultural Contexts. Cham, Springer Nature Switzerland AG: Springer International Publishing. 81–102.

[ref43] Snyder C. R. (2002). Hope theory: rainbows in the mind. Psychol. Inq. 13, 249–275. doi: 10.1207/S15327965PLI1304_01

[ref44] Stajkovic A. D. Luthans F. (1998). Self-efficacy and work-related performance: a meta-analysis. Psychol. Bull. 124, 240–261. doi: 10.1037/0033-2909.124.2.240

[ref007] Steg L. Bolderdijk J. W. Keizer K. Perlaviciute G. (2014). An integrated framework for encouraging pro-environmental behaviour: the role of values, situational factors and goals. J. Environ. Psychol. 38, 104–115. doi: 10.1016/j.jenvp.2014.01.002

[ref008] Stern D. I. (2000). A multivariate cointegration analysis of the role of energy in the U.S. macroeconomy. Energy Econ. 22, 267–283. doi: 10.1016/S0140-9883(99)00028-6

[ref45] Udall A. M. de Groot J. I. M. De Jong S. B. Shankar A. (2021). How I see me—A meta-analysis investigating the association between identities and pro-environmental behaviour. Front. Psychol. 12:582421. doi: 10.3389/fpsyg.2021.582421 33796041 PMC8008126

[ref46] United Nations Economic and Social Commission for Asia and the Pacific (2024) Asia and the Pacific SDG progress report 2024: showcasing transformative actions. United Nations ESCAP. Available online at: https://www.unescap.org/kp/2024/asia-and-pacific-sdg-progress-report-2024 (Accessed March 11, 2026).

[ref47] Van Valkengoed A. M. Abrahamse W. Steg L. (2022). To select effective interventions for pro-environmental behaviour change, we need to consider determinants of behaviour. Nat. Hum. Behav. 6, 1482–1492. doi: 10.1038/s41562-022-01473-w 36385176

[ref48] Wolde B. Lal P. Harclerode M. Rossi A. (2019). Comparative optimism: relative risk perception and behavioral response to lead exposure. Environ. Manag. 63, 691–701. doi: 10.1007/s00267-019-01148-9, 30877367

[ref49] Zawadzki S. J. Steg L. Bouman T. (2020). Meta-analytic evidence for a robust and positive association between individuals’ pro-environmental behaviors and their subjective wellbeing. Environ. Res. Lett. 15:123007. doi: 10.1088/1748-9326/abc4ae

[ref50] Zhang J. Cao A. (2025). The psychological mechanisms of education for sustainable development: environmental attitudes, self-efficacy, and social norms as mediators of pro-environmental behavior among university students. Sustainability 17:933. doi: 10.3390/su17030933

