# Supplementary material for: Efficacy and resilience are associated with environmental action: an exploratory study of psychological capital components
Source: Front Psychol. 2026 Mar 27;17:1784390. doi: 10.3389/fpsyg.2026.1784390 (PMC13065692; doi:10.3389/fpsyg.2026.1784390)
Supplement: Supplementary file 1 [file Data_Sheet_1.pdf]

Focus group assignment: Linking personality to the SDG's

Based on your experience and common sense, provide a one page (!) note where you consider the significance of each PsyCap factor in SDG terms. Will any of the PsyCap factors be more important than others? How can PsyCap relate to one or more of the SDG's?

FOCUS GROUP (FG) 1:

We think that all four of the PsyCap factors can be seen as related to the SDG, the question is to what extent and in which direction. We propose that a person with higher scores for each of the PsyCap factors will have a more solid foundation to work to achieve the SDGs (Maybe except for optimism, which can go both ways). As a group, we have gone through each of the PsyCap factors individually and discussed how they relate to the SDGs.

Firstly, *hope* is related to the SDGs because this can be seen as forming the whole basis for the SDG project. Without hope, a person would be without positive motivation, targeted energy and clear goals, which makes the creation of such an ambitious project in itself very difficult. Next, *efficacy* concerns the ability to mobilize motivation, cognitive resources and behaviors to perform specific tasks. This is related to the SDGs because to be able to achieve the SDGs, you must think that there is a possibility that you will be able to do it. However, concerning how efficacy relates to the SDGs we propose that there might be differences concerning how efficacy works in group compared to efficacy in individuals. We discussed that experts in a specific field, for example climate change, more often have a greater amount of efficacy specific to that field than laypeople. Therefore, it might be that on an individual level, efficacy is not as important for the SDGs as efficacy on a group level, since it is harder for an individual person to believe in being able to mobilize what is needed to perform specific tasks. Third, *resiliency* means a person's ability to grow and deal with injustice etc., and to be able to progress and increase responsibility. We think that this is highly relevant for the SDGs because this is a large project that will undoubtedly be met with challenges, and highly resilient people will to a larger extent be able to meet these challenges and continue moving forward with the project. Finally, *optimism* is the pattern where a person tends to explain positive experiences on personal and permanent conditions, while attributing negative experiences to external, time-limited and situational conditions. We think that optimism as a factor is relevant for the SDGs in at least two different ways. One the one hand it can be positive to have a highly optimistic person working towards the SDGs because they will be able to draw motivation from the positive things happening while not considering the negative experiences as inside their control. In this way, it can be easier to keep going and to

keep working towards what you already are doing. On the other hand, it could be unfortunate if highly optimistic people think that *everything* negative that happens is something external and situational and not related to themselves because this can cause people to not consider the world they live in and the effect they have on it, which is not the right attitude to have for example when it comes to climate change. In summary, we think all the PsyCap factors can be seen as significant for the SDGs, and it is difficult to say which is the most important. Therefore, we are saying we should take into consideration all four factors, but also that we have to be weary of the potential negative impact that we pointed out with optimism.

When it comes to how PsyCaps relate to specific SDGs, we have chosen to look at each of the factors in relation to SDG 14 (Life under water). In relation to SDG 14 hope is significant because the ambitious goals of the SDG require focused and targeted energy by both individuals and groups (for example NGOs and nations). For example, nations must change their legislation to reduce pollution and dumping waste in the oceans, often in opposition with business forces. Secondarily, if individuals take active choices, for example by preferentially choosing the products of companies that are environmentally conscience with regards to the utilization of the oceans, this will give economic incentive to work towards SDG14. Hope, being in part the presence of a clear plan to achieve a goal will likely motivate individuals to make these choices. We also wish to highlight the importance of resiliency in reaching SDG14. For individuals, resiliency might be of less importance as setbacks and mistakes generally do not affect the individual directly. However, resilience is of great importance when dealing with groups. For example, nations trying to achieve SDG14 on the international stage will likely face opposition from business interests and nations whose economies are dependent on the current usage of ocean resources. Setbacks should be expected in international diplomacy where interests are split, and the ability to deal with them and increase responsibility will likely be an important factor.

FOCUS GROUP (FG) 2: In this assignment we are trying to look at synergies between the Psychology Capital and the Sustainable development goals. The factors of Psychology Capital are hope, efficacy, resilience and optimism. We start off by looking at the first factor which is hope. Without hope that we can reach the goals, there cannot be motivation. Without motivation there will not be the required effort, and hence, there will be no progress. This is closely linked to optimism. With hope, without optimism, there cannot be a foreseeable future

worth fighting for. When it comes to efficacy, we noticed that this is quite lacking in the sustainable development agenda. Due to the voluntary tone in the whole agenda, there might be a lack of commitment to the goals and the consequences for not achieving the targets are none. For example, politicians might be more concerned about getting reelected rather than taking the necessary actions to work towards the goals, should the methods be unpopular. This is the difference between encouragement and mobilizing, and that may be the reason why progress does not match ambition. The last term in the PsyCap factors is resilience. The local policymakers need to stand in it when the population is reluctant to change their way, instead of caving. In the city of Trondheim there was a public outrage when the municipality decided to close a lot of parking lots in the city center. The public concern was that there would not be any space to park their car, but the city council stood their ground. The result: Studies show that the stores are more frequently visited, people have started walking and riding bicycles more and the people are generally happier in this part of the city. This shows the effect of being resilient, and perhaps what governments should be doing when acting on their commitments to the sustainable development agenda. To conclude, all the terms in PsyCap can relate to the sustainable development agenda. After discussing in our group, we concluded that efficacy and resilience may be the most important factors if we are to achieve the goals, but ironically, those are the scarcest to find in the stakeholders who hold the power. For us, learning PsyCap gave us a better understanding of what can we look for in terms of resources to develop. Knowing how it works, the psychology behind the SDGs, we can make some changes and face the challenges.
**FOCUS GROUP (FG) 3:** All the different PsyCap factors are important in relation to the Sustainable Development Goals (SDGs), but in different ways. The Sustainable Development Agenda, from the United Nations, consist of seventeen SDGs and 169 targets. In the following, we discuss how the terms efficiacy, hope, resiliency and optimism as presented by Luthans, Youssef, and Avoloio (2007), relate to the Sustainable Development Agenda.<br><br>Efficacy:<br>The term “Efficacy” refers to when people believe in being able to mobilize the motivation, cognitive resources and behaviors required to perform specific tasks. In relation to the SDGs, efficacy is important in several ways:

- Important on UN-level, but maybe even more important on country-level, because the countries cannot take part in an international deal without believing that they will be able to put in the work and accomplish the required goals. The UN can establish a framework based on hope and optimism, but it is up to the countries' governments to believe and see through that the tasks are completed.
- In some ways it's the most important one, because it is more realistic oriented.

Hope:

"Hope" is a state of positive motivation where people experience having targeted energy and clear plans to achieve their goals. Hope is important in several different ways in relation to the SDGs:

- The SDGs in general are based on the hope for a better world. Without positive motivation and clear plans to achieve the goals (SDGs), the core of achieving the goals would not be present. Thus, hope is an essential element for achieving the SDGs as it establishes a ground for developing the plans needed to achieve the goals that are set.

Resiliency

The term "Resiliency" refers to people's ability to grow and deal with injustice, conflicts, mistakes or even positive events, progress and increase responsibility. This term is important in relation to the SDGs in the following ways:

- Resiliency is also important when facing obstacles and negative critics. In trying to accomplish the goals, countries might face difficulties. Thus, to be able to develop and to meet the goals, countries must be able to meet obstacles on the way to increase responsibility in meeting the SDGs.
- In addition, to take responsibility of the mistakes that have been done in the past is important to learn from so that we can make a change in the future.

Optimism:

The last term, "optimism" refers to an attribution pattern where people explain positive experiences based on stable, personal and permanent conditions, while they attribute negative experiences to external, time-limited and situational conditions. Optimism is important in relation to the SDGs as:

- The SDGs are developed with a very optimistic point of view, which is important in order to push the countries to doing the best they can. However, too optimistic goals can seem impossible to accomplish and therefore undermine the purpose of creating optimism, but rather make the targets seem impossible to reach

Conclusion:

Although all these factors are an important base for international cooperation on organizational and country level, they will not make you able to accomplish the goals. Hope might be the most important starting factor for encouraging to be able to accomplish the goals. Nevertheless, all the factors are foundational in relation to the SDGs.

FOCUS GROUP (FG) 4: Hope:

Hope is first and foremost a very important factor for everything a group or an individual wishes to achieve. In this manner hope can be linked to all the SDG goals as without this attribute there would not be any goals to begin with. The fact that a big part of the world has come together to outline the SDG goals proves that hope is an important factor. To be even more specific, goal 17 demonstrates that countries that have agreed on the SDG agenda need to cooperate and help each other to create a better world. Hope is the base of these goals and ties all together as without it there would never be a vision similar to that outlined in goal 17 and the agenda as a whole.

Efficacy:

The definition of Efficacy is when people believe in being able to mobilize the motivation, cognitive resources and behaviors required to perform specific tasks. We argue that the definition of this attribute in of itself argues for how it can be tied to the SDG agenda and goals. This is because to achieve the goals set by the UN people need to mobilize their collective motivation and cognitive resources to perform the specific tasks needed to achieve said goals.

Resilience:

Resilience can be directly tied to the SDG goals, specifically goal 13. This is because even though we, the people of this world, try our best to combat the climate and reduce carbon emissions, they will rise in the coming years. This doesn't mean that our common efforts are pointless or wasted, but rather that we need to keep on fighting. We therefore need to show resiliency to achieve the SDGs.

Optimism:

Lastly, optimism is an attribute we can divide into realistic and unrealistic optimism. We need to keep in mind that to do something, we need to have the realities of life in the back of our minds. Thus, it is important that our optimism is realistic, so that the progress we make seems fair and worthy.

Summary:

Based on our group discussion we have arrived at the conclusion that the different attributes presented to us through Psychological Capital can be linked to different Sustainability goals and targets presented by the United Nations. Through a combination of all four aspects, we can lay the groundwork for the inspiration and implementation of the SDG goals.

For example, we argued that Optimism and Hope play an important role in facilitating the goals in the first place. Without any hope or optimism about the future state of the world and what we can collectively achieve to combat the problems we face there would never be any Sustainable development agenda or goals to begin with. When it comes to Resilience and efficacy, we have argued that they play an integral role in implementing policy and measures to achieve the goals that have been inspired by our collective hope and optimism.

FOCUS GROUP (FG) 5: Consider the significance of each PsyCap factor in SDG terms.

<u>Hope:</u> Hope is the pillar for action. It is represented in every SDG – they (the UN and political leaders) wouldn’t have come up with these and set them up for themselves as goals if they/we thought there was no hope of achieving them.

<u>Efficacy:</u> If you believe in yourself, that gives you the power to achieve goals. You need someone who knows their field (experts) – someone to lead who knows what they’re talking about.

<u>Resiliency:</u> We’re not being taught to be resilient enough in terms of SDGs: “we don’t have enough time!”. One problem with resilience in terms of the SDGs is that we need time to change things, but at the same time we’re being told we don’t have enough of it, which kills resilience as well as hope.

Optimism: We must adjust our expectations to be realistic – to be optimistic enough, but not based on false premises ("false" or misguided optimism). Optimism is mostly about reflecting on past actions, and that helps you better tackle future goals.
Regarding the terms of optimism and hope, we struggled to differentiate the two, but this might be because they overlap a lot in their common (layperson) meaning.

Will any of the PsyCap factors be more important than others?
Optimism may be the least important factor for SDGs.
Hope may be the most important one out of the four – "hope is the pillar for action"
Between Resiliency and Efficacy, we couldn't rank them in terms of importance. We concluded that they both support each other.

How can PsyCap relate to one or more SDGs?
Optimism can be linked to SDG number 17 especially, since it's about implementation – but of course it goes for all of them.

SDG 13 is the only one that uses the term "urgent", which means that efficacy is important. Why? Because to do something urgently you need science and knowledge of the topic or field, as well as confidence in said knowledge, which you possess.
In general, all four (hope, efficacy, resiliency and optimism) are relevant for all SDG goals, but it may differ which one is the most important one for each goal.

FOCUS GROUP (FG) 6: We discussed the different elements in the psychological capital model; Hope, efficacy, resilience and optimism, and how they are significant in SDG terms.
Hope
Hope is essential in achieving the SDG goals. It is important to have targeted energy to reach goals. If you don't have hope, there is no point in even having a goal at all. It will just be meaningless. For instance, if you don't hope that we will manage to eradicate all poverty, there is no point in listing that as a goal. On the other hand, hope is not enough alone, for instance if it's not politically possible.
Optimism
Compared to hope, we found that optimism is less important. It is important to have a realistic view. When we talk about optimism it is easy to be overoptimistic, and that could

give a false hope, which will negatively impact the reaching of the SDG-goals. If you become too optimistic you will become less eager and ready for action than you should be to achieve the goal. It can also mean that you will blame external factors, and not be motivated to make a change in behavior yourself.

Efficacy

Efficacy can be to gather tools to achieve your goals. It is to believe that you will make a change, and trust that you will find a solution. We think that efficacy is more important on an individual level, because you must believe that you can make a difference. We found that efficacy is less important what talking about the systemic or organizational level.

Resilience

Resilience might be even more important, because without resilience it is difficult to get things done on your way to achieving your goals. Resilience is to not give in even though you face a huge challenge, and the ability to work through things. You should stay focused on your tasks because issues are at stake.

In conclusion, we would like to add that all the factors are important in different ways. The capital model can be used to assess how to handle the SDGs, but there are also other tools that should be considered, for example politics. Change should happen both individually and systemically in parallel. You must believe that it is possible to change behavior. If you do something you think is good, it could mean that you easily settle and lose motivation to do anything else. But to change behavior we should make sustainable solutions the easier option and then make sure that the effect of the changed behavior is visible, in order to motivate even further.

FOCUS GROUP (FG) 7: Our Hope scores were consistently high, between 4 and 5. We think that it is important to have hope in sustainability, as you need to be able to visualize a future to work towards it. For example, the most important challenge of the SDGs is SDG1, to end poverty in all its forms. Financial security allows you to plan; without it, both individuals and countries cannot plan or work towards sustainability, as they are focused on their immediate needs.

Our efficacy scores were polarized, either low or very high. We found that the main difference was how we approached the situation of working with superiors, or people that know more than you. Some found it intimidating, while others viewed it as a learning

opportunity. This relates to SDG17 (Partnerships), as to achieve the rest of the SDG targets, experts from many different fields and countries will need to cooperate. Each expert will need to be both confident in their own knowledge, and open to others' perspectives and ideas.<br>Everyone's resilience scores were quite high. This is good as the course is unique with many unknowns, so each student will need to be confident to tackle the challenges that come their way. We believe that resilience is the most important PsyCap factor relating to the SDG Agenda, as the agenda itself is based on resilience. The world itself is not resilient and cannot sustain the current way that humans live. However, to achieve a sustainable future, we will need to overcome many setbacks, so those working towards this goal need to be prepared for any difficulty.<br>For the most part, our optimism scores were high. Optimism is important to the agenda, as the ambitious future that it hopes to achieve is a good future that can be reached if everyone takes collective responsibility. However, we did not find that the second part of the optimism definition fit all the SDG targets. For example, in SDG13 (Climate Change), we agreed that to resolve the current situation, we must take responsibility and work hard. On the other hand, if the targets are not met within the time frame, this would not be merely a result of external circumstances. Our failure would also be our responsibility, as climate change is a human-caused issue and we must correct the shortcomings of our predecessors, such as the Millennium Development Goals.
**FOCUS GROUP (FG) 8:** It is often easier to dream than to achieve if we have hope without optimism. This relates to Sustainable Development Goal (SDG) 17, which is the implementation of the goals. Based on our PsyCap analysis, we discovered that there seems to be a disconnect between hope and optimism, despite their dependencies and their shared traits that allow us to achieve. The SDGs are limited to 2030, and they are conditionally based on different world and local realities, as well as the resiliency needed to right the wrongs of our world's injustices.<br>Hope needs context. Without context hope is a mere dream. It is in human nature to hope, and it is a necessity, or else we would not have anything to live for or aspire to. It is a futuristic motivator that can have either a positive or negative outcome, but as humans we always tend to prefer the latter, to give our life meaning and substance. World complexity often dampens hope, or dilutes goals based on local priorities and what is deemed as important. The dilution

of hope opposes goal 17 of the SDGs, because implementation is the optimistic step towards our hope for the future of the planet.<br>Optimism provides the necessary guidelines or steps to achieve goals and thus, hope. Optimism provides the context for hope, but often a disconnect exists between the two due to many factors, which include: bureaucracy, individual and local situations, and various institutionalized structures. Optimism is attributional and personal, whereas hope is a broader outlook on life. Despite their separate distinctions, optimism is needed to achieve hope and exists more in the present to allow us to achieve hope which exists in the future.<br>All the SDGs, except for goal 17 relate to hope. Goal 17 is the optimistic step towards achieving all the previous 16 goals, which is where the disconnect between hope and optimism exists. The SDGs are ambitious goals; however, they can only be achieved if the implementation of the goals is done in a structured way. It is not always easy to see the silver lining in a generation that has been taught to question rather than merely to blindly accept "the truth" that comes from the top of the social structure. It is also often easier to see the outcome of goals rather than the necessary steps to achieve the set goals. It is often easier to dream than achieve, which is why we need both a bottom up and a top-down control in society, so that goals can be achievable and accessible for all, such as SDGs. The 'top' is dependent on the 'bottom' and vice versa, therefore for implementation to work and to be achievable there needs to be a connect and equal collaboration in achieving the common goals for a better and more prosperous future.<br>In conclusion, the SDGs will create an ideal world, and they are the perfect solution to right the wrongs of worldwide injustices, however this will only be achievable if we find a clear connection between hope and optimism. The most obvious way to connect the two would be to find an integrated and inductive approach to allow for small and local scale goods to be integrated upwards to a global scale, which would provide the optimism to achieve the hope of a better future and a healthier global environment.
**FOCUS GROUP (FG) 9:** The sustainable developmental goals are focused on creating a more peaceful, equal and sustainable world to meet our future challenges. The ambitious solutions to these challenges need people that are resilient when meeting with opposition. In this assignment we will be discussing how human recourse capacity can be applied to SDG13: take urgent action to combat climate change and its impacts.

Hope:

- Hope as positive motivation is important to have when faced with great challenges. The SDG plan is creating hope when countries commit to achieve the common goals. However, the SDGs are not making clear enough plans that make it possible to execute.
- Having hope is important to focus and redirect your energy towards the right goals.

Efficacy:

- Being able to adapt and learn new things is important when working with climate change research as new research is coming out.
- Efficacy is an important trait to channel your energy into something effective and productive.
- Example: being able to change your standard of living to make live more sustainable.

Resilience:

- Many climate change studies show that we must take very urgent action to combat climate change. Some use a discourse that can be deterministic. To keep up motivation to work towards a goal that sometimes can seem impossible can provoke hopelessness. Knowing your strengths (I.E being optimistic and hopeful even in hard times) and using the small hope that is left to keep you moving is an important resource to have.
- Example: when working with climate change mitigation resilience is an important tool when faced with resistance from climate change deniers.

Optimism:

- Knowing that it is possible to make a change is important to keep motivation
- However not letting optimism restrain you from taking action and making you less motivated. Optimism can decrease our sense of urgency, and we should find an equilibrium.

The Psycap specter is a good tool to analyze human resource capacity but is lacking in its missing emotional and moral aspect. We believe it is important to be resilient when working with climate change related issues.

FOCUS GROUP (FG) 10: Our group had similar total PsyCap scores, but with some variation in which factors we scored higher or lower. This was reflected in the fact that we

at times also had different perceptions on the significance and importance of the PsyCap factors as they (can) relate to the SDGs, but through our discussion we arrived at the following group consensus on the factors individually:

Hope: Without it, why even bother?

We arrived at the conclusion that goals, fundamentally, are something we want and hope to achieve. For the SDGs (or any goals) to have purpose, one must have the hope that change and/or achievement of the goal is possible. Hope functions as motivation to work towards a goal, however, we also feel that setting clear goals (and achieving them) also enforces hope and may make it stronger.

Efficacy: Belief in one's ability to achieve a goal and the ability to see solutions is a necessary fundament for effective goals and goal achievement in the same way that hope is. The ability to attribute problem solution to something we can control is also important as it relates to optimism and belief in goal achievement and prevents apathy in the face of a problem outside of our control. On a global scale, we argue that one could view the SDGs and their many targets as tools to achieve efficacy in relation to global sustainability issues.

Resiliency: Both in a human psychological context and a biological one, resiliency is the ability of a system to react to and move past shocks – to grow and deal with hardships. We discussed whether the SDGs themselves could be seen a proof of human resilience – a clearly stated vision and a plan to face the circumstances threatening the sustainability of our little blue planet. We also believe that the SDGs may help to build resilience in that the more targets and goals we reach, the more resilient our systems can and will become.

Optimism: Optimism is an important factor in our ability to envision goals and strive to reach them. However, we also discussed whether the tendency to attribute certain issues to factors outside of our control may make us lose hope, and whether ungrounded optimism may make us blind to realities we need to face, by creating false hope.

Summary: All in all, we agree that the different PsyCap factors all can relate to the SDGs to a greater or lesser effect, but that hope and efficacy may hold some more importance as these factors allow us to imagine and work towards the future that we want with the belief that we can achieve the goals we set.

FOCUS GROUP (FG) 11: The term 'Psychological Capital', also known as PsyCap, refers to a person's positive psychological developmental state and is considered a skill that can be trained and developed over time (Luthans 2007). Linking PsyCap to the SDG's may

provide useful information about how human resource capacity can help to achieve SDG's. PsyCap consists of four factors: hope, efficacy, resiliency, and optimism. The following paper will attempt to link these factors with the SDG Agenda and discuss their importance in reaching the SDG's.

HOPE

The sole existence of the SDG 2030 agenda is a manifest of hope. The agenda is an effort to channel the global community's energy with a clear set of targets towards a better and more sustainable world. Hope is required for action. There is no point in creating goals for which you have no hope of achievement. To gain full effect, hope must be integrated into the mindsets of the people and ascend from high-level policymakers to all stakeholders such as nations, organizations, and individuals. If not, the agenda will simply remain a document with unfulfilled words and promises. Therefore, we deem the PsyCap factor to be implemented in every single SDG and it will be of importance in achieving the SDG's.

EFFICACY

In our opinion, efficacy is one of the most important PsyCap factors when it comes to implementing the SDGs and the Agenda 2030. The belief in being able to mobilize to take the necessary actions for the SDG's is essential for both nations, organizations, and individuals. Efficacy refers to the stakeholder's perception of their own capabilities to implement the measures to reach the goals. To reach the targets, necessary skills and mindset must be adapted by all stakeholders. Without efficacy, the agenda may be perceived as unrealistic. To increase efficacy, people should be educated in related topics, to mobilize motivation to fulfill the goals and accomplish tasks, as mentioned in several targets.

RESILIENCE

The importance of resilience may not be directly visible in the SDG agenda. However, nations and individuals' ability to grow and deal with both positive and negative consequences is a crucial factor in the SDG agenda. When implementing the agenda in the different countries, there are going to be different levels of resilience between the

stakeholders. That may be why this factor is the most controversial, as both the nations and individuals included in the agenda vary so much in their degree of resilience.<br><br>**OPTIMISM**<br>The SDG agenda is very optimistic. This factor may be interlinked with the three factors above. Hope and optimism go hand in hand: with optimism comes hope and without hope, there can't be optimism. However, optimism must be in accordance with the agenda's efficacy: if there is too much optimism and too little efficacy, there will be a lack of, or loss of efforts. Hence, the degree of efficacy and optimism must be aligned. Lastly, resilience is increased by the amount of optimism. When the stakeholders perceive themselves as being capable of dealing with both setbacks and progress, this will increase optimism and vice versa.<br><br>In total, all the PsyCap factors are important and interrelated to ensuring global sustainable development. Some may be more relevant in certain SDG's and targets than others, but they all have a significance in implementing and achieving the SDG's. The importance PsyCap will have for the SDG 2030 Agenda remains to be seen.
**FOCUS GROUP (FG) 12:** Psychological Capital, PsyCap, is a person's positive psychological developmental state. This can be trained and developed. PsyCap consist of four factors: hope, efficacy, resiliency and optimism. Hope is motivation where people have clear plans to achieve their goals. Efficacy is about people believing in mobilizing motivation to do specific tasks. Resiliency is the ability to deal with conflicts, injustice, mistakes and positive events. Optimism is when people explain positive experiences based on stable, personal and permanent conditions, while negative experiences are explained as time-limited and situational conditions.<br>We got the task to consider the significance of each PsyCap factor in Sustainable Development goals (SDG´s) terms. Hope is an important part of the SDG´s. The SDG´s are based on the hope for a better future, for the nature and the human being. Hope is the spark; everything starts with the hope for change and then out of the hope you act.<br>If you are hopeful, you will believe that the small actions you can take makes a difference. Efficacy is important regarding to the SDG´s because of the believe that we can act. If

people did not believe that we can do something to change the world, there would be no SDG`s.<br>The latest climate reports, like the IPPC, have been extremely negative. Then it is important that the people working with the SDGs, and especially SDG 13 and 14, are resilient and continue their work even though they experience setbacks. Here resiliency comes in. in one way you can say that the SDG´s are made from people wanting to deal with injustice in the world.<br>The last PsyCap factor, optimism, is important to keep working on the SDG´s. Even when the climate report does not seem that achievable, it is important to also see the good things happening. When something turns out not as we planned, it is important not to give up but rather find a different way to solve the problem. The SDG´s may be too optimistic. Too optimistic goals can be unrealistic, and people will lose the hope in achieving the goals.<br>We think that hope is the most important factor to the SDG´s. To get things started we need hope to make progress.
**FOCUS GROUP (FG) 13:** In 2015, the United Nations set out 17 goals as a part of their 2030 agenda for sustainable development. These goals target challenges faced by the collective global society, particularly grouping targets into groups pertaining to people, planet, prosperity, peace, and partnership. Psychological Capital (PsyCap) is the term used to describe a desirable, positive psychological development state. The factors of hope, efficacy, resilience, and optimism are often considered when assessing PsyCap. Although PsyCap is usually applied to human resources and managerial contexts, the basic principles could be useful when considering the sustainable development goals. Hope: describes the positive motivation associated with when people have clear and targeted plans by which they can attain their goals. The agenda is a tool to instill hope as it lays out a targeted plan by which societal injustices can be combated. The factor of hope as an initial state is needed to act when addressing the various development goals. Hope is seen as an infectious characteristic in the human species and the concept of hope inspiring hopefulness is a concept which surpasses cultural boundaries, making it a useful tool in achieving these goals. Efficacy: is the factor that is targeted when people can mobilize motivation, cognitive resources, and behaviors to perform tasks. Mobilization of individual and collective skills is important to consider when addressing the issues currently faced by society. Regarding the SDGs, numerous targeted goals can be achieved using this factor.

Many of the goals specify the need for international cooperation to expand innovation through specialized skillsets (SDG 13). As much of this expertise is only present in developed countries, mobilization of cognitive and coordinating recourses may be needed when fulfilling the SDGgoals on a global scale. Resilience: is the ability to tolerate and grow under conditions of conflict and injustice. The fact that previous United Nation Agendas were not attained, the need for collective resilience is of the utmost importance. It is likely that the pursuit of the SDG-goals will meet comprehensive resistance from parts of the private sector, political parties, and the public. The drivers of the realization of this agenda therefore need resilience to persist when others work against the agenda. Resilience is also key to continually developing new ideas and solutions even in the face of failures, technical difficulties, and economic shocks. Optimism: can be used to describe the pattern that arises as an attribution to perception of experiences based on stable, personal, and permanent conditions. This factor may be the PsyCap factor, which is the least important to the application of attaining the SDGs. An idealistic mindset could be counterproductive when attempting to make this agenda and it may be more beneficial to have a realistic approach. Overly optimistic mindsets can lead to long term reduction of other factors such as resilience and hope
**FOCUS GROUP (FG) 14:** In the following assignment we are going to go through the four factors of the psychological capital, and their relations to sustainable developmental goals. The first factor is efficacy. It’s about how you meet a challenge, and your attitude to a task you are given. Efficacy can be useful in connection to the SDG’s because if you provide countries and people with information and skills, it will help them conquer the issues they are facing. The SDG´s are very ambitious and can seem overwhelming, especially if you don´t feel like you know how to solve the problems. Therefore, it is important to equip every country with the skillset, data and information to achieve its goals. If people get more knowledge, it will also help them get more confidence to handle bigger problems, which will provide them with even more knowledge, and you achieve a healthy spiral. Little by little everyone can contribute to the bigger picture.<br><br>Hope is a light to lead the way to a better world. As mentioned, the goals can seem overwhelming. If you believe the future is pre-determined there is no point in working towards the goal. Therefore, you need hope to believe you have the power to influence the

outcome. If the countries don't believe there is a way to reach sustainable development goals, there is no point in starting.

Change is difficult as well. Even if you have started to work towards the goals, you need resilience to be able to cope with the difficulties related to change. The SDGs are set to a specific date, and the closer you get to 2030, the more stressful the goals will become. You also need to be able to adjust your plans after facing challenges, it is a trial-and-error process. This requires resilience.

Again, reaching goals is an ambitious process, and optimism is needed to be able to act on the beliefs that you can change society. Optimism is to look at the problems as temporary and possible to fix.

When we discussed which of the factors were more important, all of us concluded differently, and all four factors were mentioned. Resilience was mentioned because there is important to keep going. An example is that to reach our goal on climate change, in Norway we would have to change our way of living, like eat less meat, lower our consumption of clothes and electronics. To be able to stand in these changes we need resilience. Hope was mentioned because if you don't believe you can do something about a problem, there is no point in starting. Optimism and resilience were mentioned in a combination, because when you start something, you need to be able to finish it, and when things get hard, you cannot drop out. Efficacy was mentioned because if you have the means and the skills to solve a problem, hope and optimism will come with it. In the end, Shuda concluded with none of the factors being more important than the other and explained it with a metaphor. The four factors represent the four legs of a stool, and all of them are needed to keep it in balance. Since we all mentioned different factors as the most important, this shows how all of them are equally useful to reach sustainable developmental goals.

FOCUS GROUP (FG) 15: Hope is important to create motivation and is essential to create engagement and effort. Hope is essential both for larger groups but also individuals. The importance of individual hope is huge, considering every small act helps. People need to see that their efforts are contributing and the different ways they can contribute. For a large

group, hope is essential to create a sense of togetherness and unity, a feeling of working towards a common goal.

All the PsyCap factors are highly relevant for the SDG terms, each one in their own way. Efficacy, whitch is about presenting work and contributing to discussion and presenting information, is important because the SDG terms need to be talked about and needs awareness-raising. We need to contribute with feedback and be able to keep track of the progress that is important during the entire process to effectively reach the goals.

Resilience is important because handling stress and challenges in the right way is key in these 14 yearlong plans about sustainability. The ability to work well when meeting unexpected events and adapt to and adjust to difficult situations is essential if the entire world is to reach all the goals. To stay calm and focused is especially important considering we are working towards goals for fixing problems that are constantly changing and growing, with a time limit as well.

Optimism is important as well considering this process is long, over 14 years, and the improvement will not always be constant, sometimes things will seem slow and then it is important to keep the optimism up. The solutions aren't always coming right away either and therefore optimism is important to keep the good spirit while we watch the solutions come alive.

Efficacy is important to create results, but to work smartly we need to be motivated, which comes from hope. On the other hand, hope is not always enough; we need resilience as well during though and challenging times. And to be able to work in hard times optimism is important to make things happen. To conclude, every PsyCap is important in its own way, but we believe every one of them relies on the others to be a good tool. HERO works in the same ways as the SDG's we believe. Every single one is important but will not work without the others.

FOCUS GROUP (FG) 16: Psychological Capital (PsyCap) is a measure of a person's positive psychological resources. Increasing an individual's or group's PsyCap will increase the individual's or group's performance and productivity. The term stems from the field of

social psychology and consists of four factors: Hope, Efficacy, Resilience and Optimism, which are evaluated based on a set of questions rated from 1-6. PsyCap is a useful tool often used by for example company managers to evaluate their employees' capacity to fulfill their work. Increasing the feelings of hope, efficacy, resilience, and optimism in a group will result in a higher ability to cope with problems. So far, the theory of Psychological Capital has not been used on a global scale, but if done so, it could perhaps help humanity in reaching the Sustainable Development Goals. In this short overview we will try to adapt the PsyCap factors to the SDGs, in particular SDG13: taking urgent climate action.

Hope in PsyCap terms is defined as "a state of positive motivation where people experience having targeted energy and clear plans to achieve their goals". (1) Many people today suffer from "climate depression", a state of hopelessness in regard to climate mitigation.* Installing the belief that we can still make a change for the better is the first step in reaching the SDGs and crucial in creating targeted energy rather than a feeling of paralyzing negativity.* We believe this could be done by acknowledging our progress and success so far, and setting a realistic time plan with small goals. For example, coming from South Africa which runs 90% on coal, it seems impossible for a whole country to run on green energy. Hearing that Norway is running on 95% hydropower shows that it is possible and gives hope for the transition toward green energy. There are many other examples which show that humans are better at change than we realize. Changing societal norms and behaviour can sometimes be done quickly, for example the transition from smoking indoors to smoking only in restricted area was accomplished in a relatively short time. Regarding climate action, we don't see the positive changes because today there are no reporting tools (indicators) in place to continuously evaluate our progress. Doing so could install hope and measure how well we are doing.

Efficacy in PsyCap terms is defined as "believing in being able to mobilize the motivation, cognitive resources and behaviors required to perform specific tasks." (1) There are two problems here, the first being that taking climate action is a very complex topic, and the second being that many people don't believe that they have any real influence on the solution. To increase our efficacy in reaching the SDGs, we need to boil it down to specific tasks with indicators to measure progress and change our thought pattern into a group

feeling where everyone does their part. This targeted motivation (hope) needs to come from both the people and the policy makers. People need to show that they are willing to change their behaviour to reach a social tipping point which gives the policy makers enough support to take the drastic measures needed<br><br>**Resilience** in PsyCap terms is defined as "the ability to grow and deal with injustice, conflicts, mistakes […] and increase responsibility." (1) On a personal level, this can be done by remembering other situations where one has overcome difficulty and using the same tools that helped us then. On a collective level, this can be difficult because we are not all aware of difficulties overcome long ago. We believe increasing our ability to deal with climate related issues can be achieved by using tools from previous global challenges. The worlds collective ban on harmful chemicals to mitigate the hole in the ozone layer is one such example. It shows that global and targeted effort is not impossible. Of course, having steps in place for disasters, being prepared for flooding and drought also increases our societies sense of resilience.<br><br>**Optimism** in PsyCap terms is defined as "an attribution pattern where people explain positive experiences based on stable, personal and permanent conditions, while they attribute negative experiences to external, time-limited and situational conditions". (1) Optimism in this sense will not help us in reaching the SDGs. * We cannot attribute all negative changes to external factors but need to realize that we are the cause of the current unsustainable situation.<br><br>In a way, this realization will increase the other three factors of PsyCap, because if we are the cause of the problem, then the solution is not out of our control but lies in changing our behaviour.
**FOCUS GROUP (FG) 17:** The positive psychological developmental state is considered "psychological capital" (Psycap). Psycap consists of four factors: hope, efficacy, resilience, and optimism. This paper will discuss these concepts in the context of the United Nations sustainable development goals (SDG's). Focusing on why these concepts are important for the success of the SDG's.

Hope is a "state of positive motivation where people experience having targeted energy and clear plans to achieve their goals". Hope is an important tool for achieving sustainable development goals because if we do not have hope, each goal will seem too big to achieve. To maintain the drive to achieve all these goals we must hope that these goals are achievable. For example, SDG 13 "Take urgent action to combat climate change and its impacts", people tend to have a dreary view of the future regarding climate change. It is important to instill hope that climate change can be combatted, and it is not too late to make a difference.<br>Efficacy is the belief in your ability to mobilize the motivation and resources available to perform the task at hand. It is easier to continue working towards the SDG's if you believe that your contributions make a difference and are relevant. We can hope for each of the goals to be achieved but without clear targets it makes it difficult to achieve them. The targets of the SDG's provide a clear direction to guide our contributions. All the SDG's require collective contributions from all countries to be achieved. Efficacy is needed to believe that our actions and contributions help achieve these goals.<br>Resilience is the ability to grow and deal with injustice, conflicts, mistakes or even positive events, progress and increase responsibility. To achieve the SDG's, we must accept that this will require huge changes to our lifestyles and society. We need to be able to adapt, grow and deal with these changes to progress and take responsibility for the SDG's. These SDG's will take time to accomplish and without resilience they will not succeed.<br>Optimism is important for the success of the SDG's because lack of optimism reduces our hope, efficacy, and resilience. We need as much hope, efficacy, and resilience as possible to conquer our SDG's and optimism can help us achieve this.
**FOCUS GROUP (FG) 18:** The factors that constitute PsyCap, Efficacy, Hope, Resilience, and Optimism, are each indispensable to the implementation of the SDG's. This paper will discuss the importance of each factor to the SDG's and offer a specific example of a goal in which they would be needed. Efficacy mobilizes people to work together to understand an issue at hand and act accordingly. Individuals need to feel empowered to share ideas and make changes according to the SDG's. Efficacy encourages personal commitment, and a common attitude change which is necessary to accomplish goals. For example, to work towards Goal 5: Gender Equality, will require input from women and girls to share their experiences and be part of the conversation for change. Encouraging efficiency in women

and girls is therefore an essential part of their empowerment. Hope helps with motivation and engagement. It gives a feeling of purpose and understanding that what individuals are doing is meaningful and important. Without hope, the SDGs cannot be accomplished because there is no reason to accomplish them. We therefore need hope to believe that our actions matter and can make a change. The 2030 Agenda is itself extremely hopeful. Hope, in addition to a clear plan and other factors such as resilience and engagement, can lead to lasting change. For example, to accomplish Goal 10: Reduce inequality within and among countries, is a huge task and seems almost unachievable. Each country is unique, with different amounts of land, resources, money, etc. These factors will always be there and are huge roadblocks to bringing the world closer to equality. Because of this, a great deal of hope is needed to energize people to continue this effort. Resilience is important because it helps us try again after failure. SDG's themselves are resilient because the Millennium development goals were not met. The SDGs are in some way a continuation of them. Challenges will definitely be present as changes happen according to SDG's; therefore, resilience is necessary. Resilience interacts with optimism in that once we have passed one goal, we can use it as a milestone to support our optimism. For example, to meet Goal 17: Implementation, resilience is important because the implementation of any program never goes smoothly. Lastly, optimism is crucial to meet the SDG's. Without optimism, we don't think things are possible to accomplish. With optimism, however, comes some concern. While it is essential to group morale, optimism carries with it a danger of looking at the world through rose-tinted glasses. To accomplish the SDG's, a healthy level of optimism, combined with an honest look at our current situation, will be necessary. For example, to accomplish Goal 12: Ensure sustainable consumption and production patterns, optimism is necessary to fuel creativity, innovation and investment in new sustainable technologies. To summarize, each PsyCap factor will play an essential role in the journey to accomplishing the SDG goals. However, we think that the combination of hope, resilience and efficacy are the most important of the four.
